# Indents

**Published:** 1918-06

**Authors:** 


### INDENTS

☞ **Brief Articles of Technical or Scientific Value will receive notice and comment. Contributions invited.**

*Soldier Dental Mechanics* In England they are finding discharged soldiers with some mechanical ability good material for dental technicers. There is no reason why with proper training such men should not be utilized in this way to very great benefit in helping out in the great dental needs of the times.

*Denture Polishing Material* After filing, scraping and sanding, use a polishing paste made of equal parts of pumice, emery and silex of medium fine grit. If combined by a mixture of equal parts of beeswax and hard oil melted together all spattering of dust will be avoided and rapid and satisfactory polishing will be done.—F. W. Frahm, in *Pacific Gazette.* Care should be exercised to avoid overheating vulcanite plates where fast-running lathes are employed.—Editor REGISTER.

*To Avoid Air Bubbles in Plaster Casts* After coating impression with separating medium apply a moderately thin mix of plaster to the entire surface of impression with a ½-inch oval paint brush (not camel's hair). When coated, immediately immerse brush in a glass of water, and finish pouring cast in usual manner. This avoids jarring of impression with probable fracture or distortion. Allow brush to remain in water. When necessary to use it again, the plaster will shake or jar out readily and brush is as clean as when new. —Lester N. Roubert, in *Review.*

*Preparing Root End for Crown* In shaping bicuspid and molar crowns, after removing occlusal and lateral surfaces with stones, the difficult angles may be easily reached by using coarse cloth or paper disks treated with vaseline or hard oil to prevent water disintegration and to facilitate their abrasive properties. They can be made to cut around curves or angles by proper manipulation.—F. W. Frahm, in *Pacific Dental Gazette.*

*A Help in Making Inlays* When inlays are made by the indirect method there are occasionally slight irregularities in the amalgam die, which, when transferred to the inlay, prevent seating it perfectly. I have tried many plans for doing away with these, and have recently hit upon a method which is very satisfactory. So far as I know it is original.

When the amalgam die is ready for shaping the wax pattern, paint inside of die cavity with shellac and blow out the excess varnish and wipe all shellac away from the cavity margins. Set the die aside and let the shellac harden.

When shellac is thoroughly hard, make inlay as usual. Since I have been using this method, inlays have gone to place much more perfectly than ever before, and it has saved me much time and annoyance.—E. S. Ulsaver, in *Digest.*

*To Cure Sensitive Incisal Surfaces* As age advances and the incisal ends of the teeth wear down, they sometimes grow very sensitive to acid foods. Procure a styptic stick such as barbers use to stop bleeding, and rub this on the sensitive spot and the trouble will be instantly relieved. This stick is also excellent for cold sores on the lips.—James W. Cormany, in *Review.*

*Apothesine in Dentistry* Apothesine is used in dentistry in the same manner as other soluble local anesthetics. It is indicated in all minor operations and may be used successfully in many major operations upon the mucous surfaces, the gums, alveolar structure, and the teeth. Like other synthetic anesthetics, it is absorbed more slowly than cocain, and for that reason a little more time should be allowed to produce the effect by submucous, subperiosteal or peridental injection, or when injecting directly into a nerve trunk—nerve block—or in pressure anesthesia. —*Therapeutic Notes.*

*Root Canal Therapeutics* The best possible kind of root-canal therapeutics that can be practiced is the preventive kind, that kind which begins before a cavity in a tooth occurs.

In other words, we want to impress upon you people here the fact that we always have believed, still believe, and always will believe that the teaching and the practice of preventive dentistry is the most important obligation that rests upon the dental profession. It stands second to nothing in all the realm of dentistry. We are sorry to say, however, that at least nine-tenths of the members of the profession in actual practice give "reparative dentistry" first thought and consideration. We regret to say also that the men who are giving first consideration to reparative dentistry are, with but few exceptions, giving root-canal treatment or therapy the least consideration of any part of that line of practice.—W. G. Ebersole, in *Summary*.

*Vinegar for Softening Plaster of Paris* Vinegar, having the property of disintegrating plaster, may be employed in the laboratory for removing plaster from flasks, by immersing for a few seconds only; for removing casts from articulator by applying over bows only; cleaning impression trays, etc.—Lester N. Roubert, in *Dental Review*.

*Assuring the Timid* Lord Cromer tells this story of Ishmail Pasha, Khedive of Egypt. It once happened that Ishmail was suffering from toothache. He sent for a European dentist, who told him that he was afraid it would be very painful. He was informed in reply that if he would allow the dentist to administer laughing gas to him he would feel nothing. He still doubted, but told the dentist to bring his apparatus to the palace. The dentist complied and explained the process to the Khedive. Ishmail then summoned an attendant and told him to send up the sentry who was at his door. When the man arrived the Khedive ordered him to sit down in a chair and requested the dentist to take out a tooth on either side of his jaw. Ishmail then asked the man whether he had felt anything, and the man told him that he had not. But Ishmail was not yet satisfied. He said that the sentry was a young, strong man and that he would like to see the experiment tried on some one of weaker physique. Accordingly he summoned a slave girl and had the dentist extract two of her teeth. Finding that she did not show evidence of extreme suffering, he then consented to have his own tooth out.—*Dental Record.*

*Attaching Bridges to Vital Teeth*

In most cases where teeth adjacent to the tooth spaces are sound the bridge had better be made of the removable type, not only to conserve the pulps, but the teeth used for support as well. If we pretend to practice conservative dentistry, let us be consistent. I am confident we should use the removable appliances in the great majority of these cases. I know that these appliances can be made so that they are useful and comfortable, and I will take my chances on the destructive tendencies that they may have upon the natural teeth. We must use the appliances intelligently; we should acquire a broader understanding of their construction and application and their care. Instruct your patients to take care of these appliances in order to avoid possible decay around them. That is all entirely feasible and practicable and simple as compared to many of the appliances that are being used in a fixed way.—Dr. F. E. Roach, in *Dental Review.*

*Chloro-Percha Does Not Radiograph*

While doing some research work relative to root-canal fillings and focal oral infection, I was struck by the large number of root fillings I had personally placed which showed a healthy apical region with the root filling only one-half or two-thirds of the way to the apex, the remaining one-half or one-third showing as though there existed an entirely unfilled canal which appeared of easy access, yet small.

I therefore did the following experiment, which I would advise every radiographer to perform to prove up my findings:

I prepared four small, straight root canals, as found in lower centrals. I broached them with a small reamer until the broach would protrude slightly through the apical end.

I flooded the canals with eucalyptol, then pumped into the canals chloropercha as used by dentists generally for the past thirty years. This was pumped into the canal until it formed in a ball beyond the apex. A canal point was forced into the canal one-half the length of the canal, the canal point having been previously measured and cut to one-half the length of the root. The excess chloropercha was then wiped off the end of root.

These roots then were waxed lightly to the face side of a film and exposed full time, at the usual distance. The result showed nothing in the apical half of the roots, while the canal point showed up clearly for the portion it occupied. The above easily is proven, in which case I would like to ask how much does the radiograph show us about the presence or absence of root fillings placed these thirty years?

I am at present using a radiodescent chloropercha made by adding eight grains of bismuth subnitrate to each drachm of chloroform before dissolving in the guttapercha.

Dentists and radiographers better try this experiment and stop to think a bit before they diagnose "no root filling," when the apical third seems open in the Roentgenograph.—W. Clyde Davis, Lincoln, Neb.

*Pioneers in Root Resection* Dr. J. N. Farrar was one of the first American dentists to practice root end resection. In 1884 he reported the following: "Nine years of successful practice of this operation, after eleven years of ups and downs in trying to save such far-gone cases by the old-fashioned palliative treatment, warrants me in giving a decided reply in the affirmative. The amputated portion may vary from a small extremity about the apex to the entire root, depending upon the extent of its degeneration, but more generally upon the degree of destruction of the socket walls." During the same year, Dr. Geo. P. Mann, of Chicago, reported having operated on a patient for the amputation of the anterior root of the lower right second molar. Dr. P. T. Smith, in 1889, reported having amputated a molar root which was exostosed for the relief of neuralgia. He claims to have excised many molar roots for the cure of neuralgia. In 1890, Dr. M. L. Rhein read at paper before the American Dental Association and reported in the *Dental Cosmos* on "Amputation of Roots as a Radical Cure in Chronic Alveolar Abscess," in which he said that "although amputation of a portion or the whole of the root for the restoration to health of teeth which did not yield to milder treatment had long been advocated by the few, the textbooks were singularly silent about this important procedure. Usually the operation presented no difficulty, and except in the region of the antrum or the mental foramen, no dangerous anatomical points were involved. The method of treatment was to fill the root or roots, excise the diseased portion, following with a vigorous use of the bur in the surrounding pathological tissue. If the operation was performed under aseptic conditions and the parts kept so until the wound was entirely healed, an immediate and radical cure resulted."

In 1902, Dr. Gordon White reported before the National Dental Association his method of root amputation for the cure of alveolar abscess. He claims to have performed his first operation in 1886. At that time he amputated the root end of the upper lateral.

In 1902, Dr. M. L. Rhein read a paper before the Northwestern Dental Association, demonstrating his method of root amputation and replacing the lost structure with a porcelain root.

In 1905, Dr. M. I. Schamberg read a paper before the Pennsylvania State Dental Society entitled, "The Surgical Treatment of Chronic Alveolar Abscess." His operation consisted of a rapid ablation of the diseased area, including the end of the root by means of a swiftly running surgical bur. A sharply defined round opening is thus made through the gum and alveolus and the end of the root is burred away. After syringing the cavity thus produced, it is packed tightly with iodoform gauze.

Dr. A. Masm, in the *Deutsche Monatsschrift fur Zahnheilkunde,* March, 1906, reports that the surgical treatment of an alveolar abscess which consists in the removal of a portion of the apical area of the root is not warranted by the conditions encountered in such cases. He recommends a complete surgical removal of all pathological formations around the end of the root. Masm recommends a vertical incision from the fistulous opening in the direction of the apex of the root. With a round bur the alveolus is now perforated and every particle of necrosed alveolar and root tissue is by this means removed. The root is then treated with fifty per cent. sulphuric acid and the wound with a thymol solution. Inside of five days, in all ordinary cases, the wound has completely healed. Since 1906, quite a number of reports on root amputation have been published in our dental literature.—Dr. Federspiel, in *Dental Items of Interest.*

*Licenses Revoked* At a meeting held April 2, the Ohio State Medical Board revoked the licenses of Drs. Robert Austin Browne and Frank Llewellyn Bowsher, both of Akron, for extravagantly worded and dishonest newspaper advertising. May 6, for a similar reason, the board also revoked the license of Dr. Franklin Stuart Temple, formerly of Toledo. Dr. Browne had advertised "quick results" for "weak, wornout men." Dr. Bowsher had advertised himself as "Akron's reliable specialist," and Dr. Temple was proclaiming himself as a magnetic healer and had conducted a public clinic in the opera house at Lorain. Action was taken under the law which makes it illegal for a licensed practitioner to issue advertisements intended, or having a tendency to deceive and defraud the public.—*Journal A. M. A.*

